# Use of Oral Anticoagulants in Patients with Atrial Fibrillation: Preliminary Data from the Italian Atrial Fibrillation (ITALY-AF) Registry

**DOI:** 10.3390/clinpract13050105

**Published:** 2023-09-27

**Authors:** Fabio Angeli, Gianpaolo Reboldi, Giancarlo Agnelli, Giuseppe Ambrosio, Alessandro Capucci, Giovanni Carreras, Claudio Cavallini, Adriano Murrone, Gaetano Vaudo, Gianluca Zingarini, Paolo Verdecchia

**Affiliations:** 1 Department of Medicine and Technological Innovation (DiMIT), University of Insubria, 21100 Varese, Italy; 2 Department of Medicine and Cardiopulmonary Rehabilitation, Maugeri Care and Research Institutes, IRCCS Tradate, 21049 Varese, Italy; 3 Department of Medicine, University of Perugia, 06123 Perugia, Italy; 4 Internal Vascular and Emergency Medicine—Stroke Unit, University of Perugia, 06123 Perugia, Italy; 5 Maugeri Scientific Clinical Institutes—IRCCS, 27100 Pavia, Italy; 6 Division of Cardiology, School of Medicine, University of Perugia, 06123 Perugia, Italy; 7 Cardiology and Arrhythmology Clinic, Marche Polytechnic University, University Hospital Umberto I, Lancisi-Salesi, 60123 Ancona, Italy; 8 Arrhythmology Unit, Cardiology Department, Terni University Hospital, 05100 Terni, Italy; 9 Struttura Complessa di Cardiologia, Hospital S. Maria della Misericordia, 06129 Perugia, Italy; claudio.cavallini@ospedale.perugia.it (C.C.);; 10 Struttura Complessa di Cardiologia, Hospital of Città di Castello, Città di Castello, 06012 Perugia, Italy; 11 Unit of Internal Medicine, Terni University Hospital, 05100 Terni, Italy; 12 Fondazione Umbra Cuore e Ipertensione-ONLUS, 06124 Perugia, Italy

**Keywords:** atrial fibrillation, guidelines, therapy, oral anticoagulants, registry

## Abstract

Background: Atrial fibrillation (AFIB), the most frequent cardiac arrhythmia, is a major risk factor for stroke, heart failure, and death. Because of the recent advances in AFIB management and the availability of new oral anticoagulants (OACs), there is a need for a systematic and predefined collection of contemporary data regarding its management and treatment. Methods: The objective of the ongoing ITALY-AFIB registry is to evaluate the long-term morbidity and mortality in patients with AFIB and to verify the implementation of the current guidelines for stroke prevention in these patients. The registry includes consecutive in- and out-patients with first diagnosed, paroxysmal, persistent, or permanent AFIB. In patients in sinus rhythm at entry, the qualifying episode of AFIB, confirmed by ECG diagnosis, had to have occurred within 1 year before entry. The clinical record form is web-based and accessible by personal keyword. Results: Enrolment into the registry started in the year 2013. In a current cohort of 2470 patients (mean age 75 ± 11 years, males 56%), the mean CHA_2_DS_2_-VASc score was 3.7 ± 1.8, and the mean HAS-BLED was 1.6 ± 0.9. There were no significant sex differences in the AFIB subtypes. At the end of the inclusion visit and after receiving knowledge of the web-based electronic estimate of risk for stroke and bleeding, the proportion of patients discharged with OACs was 80%. After exclusion of patients with first diagnosed AFIB (n = 397), the proportion of patients with prescription of OACs rose from 66% before the visit to 82% on discharge (*p* < 0.0001). Prescription of aspirin or other antiplatelet drugs fell from 18% before the visit to 10% on discharge (*p* < 0.0001). Conclusions: A web-based management of AFIB with automated estimation of risk profiles appears to favorably affect adherence to AFIB guidelines, based on a high proportion of patients treated with OACs and a substantial decline in the use of antiplatelet drugs.

## 1. Introduction

Atrial fibrillation (AFIB), the most frequent cardiac arrhythmia [1,2], is a major risk factor for embolic events, heart failure, and death [3]. Several clinical trials and meta-analyses have shown that in patients with any form of AFIB (i.e., first diagnosed, paroxysmal, persistent, or permanent), oral anticoagulation (OAC) with warfarin significantly reduces the risk of embolic events and stroke [3,4,5,6,7,8].

It is generally accepted that anticoagulation may reduce the risk of clinical stroke by two-thirds when compared with placebo, with the expected degree of absolute benefit depending on baseline risk [6,9,10].

Because of the recent advances in AFIB diagnosis and management and the availability of new oral anticoagulants (NOACs) [9], there is a need for a systematic and pre-defined collections of modern data regarding its management and treatment.

To date, some important tools have been developed in order to assist clinicians in the process of initiating or titrating OACs by estimating the risk of stroke or bleedings [11,12,13]. Unfortunately, primary care clinicians have limited time during visits and may lack adequate systems for collecting and applying these tools. As a result, despite the availability of these tools for several years, many patients with AFIB who meet the criteria for initiating OACs still remain untreated [10,14,15].

In this setting, it is not clear whether electronic health records or web-based decision support tools may contribute to improving the correct use of anticoagulation and the adherence to treatment.

For this purpose, we present the protocol of an ongoing web-based (www.italiafa.eu, accessed on 30 July 2023) Italian registry on AFIB (ITALY-AF), and some preliminary findings on the feasibility and effectiveness of a web-based decision support tool.

## 2. Materials and Methods

ITALY-AF is a multicenter, prospective ongoing registry of patients with non-valvular AFIB.

Patients are recruited (either as inpatients or outpatients, aged ≥ 18 years) if they have had non-valvular AFIB. Non-valvular AFIB is defined by the absence of moderate or severe mitral stenosis or mechanical heart valves. To ensure the inclusion of a broad spectrum of representative patients with AFIB, a variety of outpatient practices were targeted for this registry.

To date, enrollment is being performed in a total of 22 hospitals or out-patient facilities in the setting of the Italian Health System (IHS, beginning in January 2013). The registry represents a collaboration of health care providers, including cardiologists, internists, neurologists, and electrophysiologists.

Although investigator specialty identification represents the most reproducible method to ensure site heterogeneity, geographic diversity is also considered in final site selection and patient enrollment, and for this reason we adopted an adaptive registry design. Thus, sample size may be modified during the course of the study to ensure adequacy of the registry to answer targeted research questions. Adaptive changes to the sample size for sub-studies will be also considered during patient enrollment. Patient characteristics will be compared before and after adaptive changes to ensure that generalizability is not impaired.

All patients are required to sign a written informed consent form; the registry is conducted in accordance with the EU Note for Guidance on Good Clinical Practice CPMP/ECH/135/95 and the Declaration of Helsinki. Each participating center (see Appendix A) obtained the approval of the local ethics committee.

At baseline, evaluation includes a detailed clinical examination, laboratory tests, types of AFIB, and comorbidities.

For enrollment, patients must be affected by AFIB at entry into the registry or, in the case of paroxysmal or persistent AFIB currently in sinus rhythm, by evidence of AFIB within one year before entry. Several diagnostic techniques were used for the diagnosis of AFIB, including standard electrocardiogram (ECG), ECG-Holter monitoring, and pacemaker diagnostics.

Mechanical valve, life-threatening conditions with life expectancy <1 year, and severe mitral stenosis are exclusion criteria.

Based on the presentation, duration, and spontaneous termination of AFIB episodes, five types of AFIB are distinguished: (i) first diagnosed, (ii) paroxysmal, (iii) persistent, (iv) long-standing persistent, and (v) permanent AFIB [14,15].

The presence of comorbidities and cardiovascular risk factors was defined according to documented medical history, as collected by physicians at the study site level. This initial assessment was performed by any clinicians during the clinical interview with the patient and by searching through medical records.

Baseline data on cardiovascular risk factors and treatment strategies in use before and after the entry visit into the registry are stored in a clinical web-based record form (www.italiafa.eu, accessed on 30 July 2023).

After the inclusion of each patient in the web-based form and before discharge, the following tools for assessing the benefit from stroke reduction or the increase in bleeding risk with anticoagulation are automatically calculated and displayed:

- HAS-BLED (hypertension, abnormal renal/liver function, stroke, bleeding history or predisposition, labile international normalized ratio, elderly (> 65 years), drugs/alcohol concomitantly) score to assess the individual bleeding risk of real-world patients with AFIB [13];
- CHA_2_DS_2_-VASc score for stroke risk stratification [12].

In the case of a lack of prescription for OACs in a patient who met criteria for anticoagulation therapy, a ‘warning window’ opens, highlighting the lack of compliance with treatment recommendations.

### Statistical Analysis

We use STATA 16 (StataCorp, College Station, TX: StataCorp LLC, USA) and R software version 3 (R Foundation for Statistical Computing, Vienna, Austria. URL http://www.R-project.org; accessed on 30 July 2023). We present data as mean ± standard deviation (SD) or median (and interquartile range, IQR), when appropriate for continuous variables, and proportions for categorical variables. Differences in proportions between groups are analyzed using the χ^2^ test. Mean values of variables are compared by independent sample t-test or analysis of variance (ANOVA), when appropriate. Logistic regression model is used to test the relationship between the demographic, clinical, and laboratory findings with the occurrence of OACs prescription. In two-tailed tests, *p*-values < 0.05 are considered statistically significant.

## 3. Results

**Figure 1:**
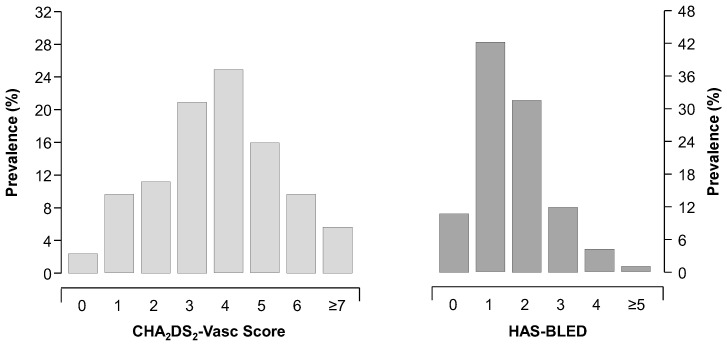
Distribution of CHA_2_DS_2_-VASc and HAS-BLED scores in the overall cohort (n = 2470).

Enrolment into the registry started in January 2013. We now present data from 2470 patients (mean age 75 ± 11 years, males 56%) recruited on 31 January 2023. Of the whole cohort, the mean CHA_2_DS_2_-VASc score is 3.7 ± 1.8, and the mean HAS-BLED is 1.6 ± 0.9 (Figure 1).

Among ECG characteristics, 62% of patients have AFIB at entry-ECG (with a mean heart rate equal to 82 ± 23 b.p.m). The proportion of male patients is 56%, and there are no significant sex differences in the AFIB subtypes (Table 1). Other main characteristics of recruited patients (including cardiovascular risk factors, comorbid conditions, and previous vascular events) are shown in Table 1.

Men are younger, more frequently smokers, and more likely to be obese than women. The estimated glomerular filtration rate (eGFR) is lower and the history of myocardial infarction higher in men than in women. Women show a two-fold increase in the frequency of prior pulmonary embolism when compared to men (2.6% vs. 1.2%, *p* = 0.010).

In the whole cohort and at the end of the inclusion visit with a web-based electronic estimate of risk scores for stroke and bleeding, the proportion of patients with prescription of OACs is 80%. The proportion of patients receiving warfarin is 58%. As expected, both CHA_2_DS_2_-VASc and HAS-BLED scores are independent predictors of OACs prescription at discharge. After multivariable adjustment, for each increment in CHA_2_DS_2_-VASc score, the probability of prescription of OACs significantly increases (OR: 1.28, 95% CI: 1.18 to 1.38, *p* < 0.0001). Conversely, for each increment in HAS-BLED score, the likelihood of receiving a prescription of OACs decreases (OR: 0.62, 95% CI: 0.55 to 0.71, *p* < 0.0001). Notably, time-trend analysis showed non-significant changes in prescription of oral anticoagulants over time (ranging from 82% in year 2013 to 80% after year 2019, *p* = 0.181).

**Figure 2:**
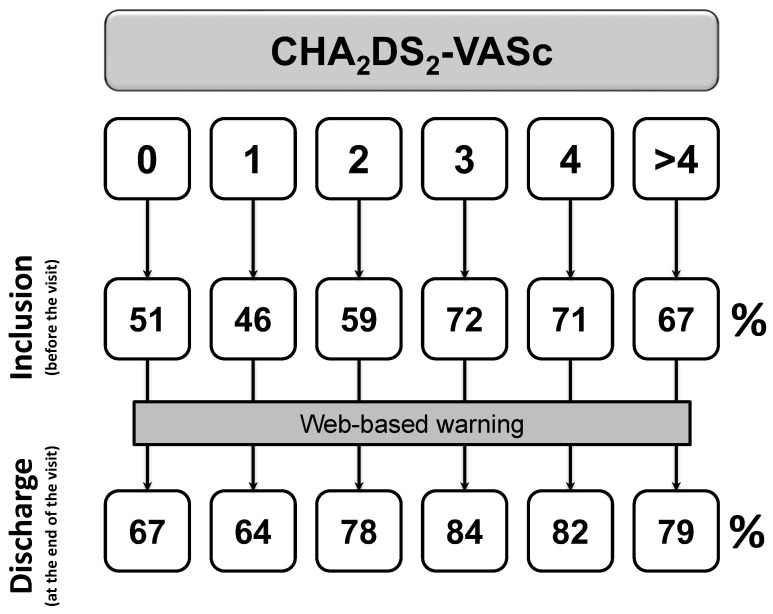
Changes in OACs prescription among patients with at least one previous episode of documented AFIB (*p* < 0.0001). Inclusion denotes the status before the visit, discharge is the status at the end of the visit.

**Figure 3:**
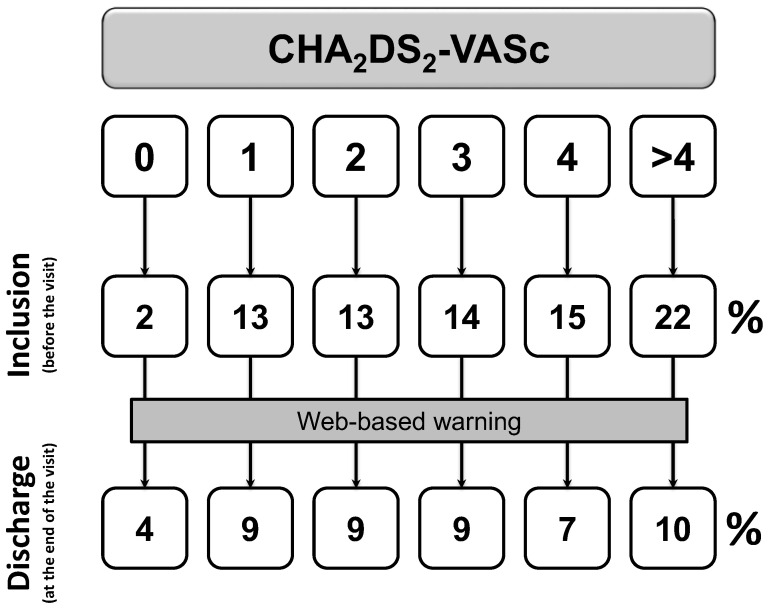
Changes in anti-platelets prescription among patients with at least one previous episode of documented AFIB (*p* < 0.0001). Inclusion denotes the status before the visit; discharge is the status at the end of the visit.

After exclusion of patients with first diagnosed AFIB (*n* = 397), the proportion of patients with a prescription of OACs rises from 66% to 82% (*p* < 0.0001, Figure 2) from before visit to discharge. Prescription of aspirin or other antiplatelet drugs falls from 18% to 10% (*p* < 0.0001, Figure 3).

Unexpectedly, among patients with CHA_2_DS_2_-VASc equal to 0, a proportion of 67% received OACs following the web-site warning (Figure 2). Similar results were also recorded for CHA_2_DS_2_-VASc equal to 1 for women. According to clinical interviews with recruiting centers (see Appendix A), prescription of OACs were mainly driven by (i) the occurrence of long-term persistent or permanent AFIB candidated to electric cardioversion (68%), or (ii) long-term persistent or permanent AFIB with an HAS-BLED score equal to 0 or 1 (96%).

On the other hand, lack of prescription of OACs among patients in whom guidelines recommended anticoagulation was mainly driven by an HAS-BLED score >2 (76%) or patient refusal (7%).

## 4. Discussion

Irrespective of the underlying risk factors and pathophysiology [10,16,17], AFIB is the most common cardiac arrhythmia, and arterial thromboembolism is its most serious complication [1,2,18].

OAC with warfarin significantly reduces the risk of stroke and embolic events by about two-thirds [6,19]. In the landmark report from the Swedish AFIB cohort study [19], the adjusted net clinical benefit favored anticoagulation for almost all AFIB patients; moreover, the risk of ischemic stroke without anticoagulant treatment was higher than the risk of intracranial bleeding with anticoagulant treatment [19].

Taken together, these findings suggest that more patients may benefit from OACs. Although the recent availability of NOACs, which have been proven to be associated with a lower rate of hemorrhagic strokes and intracranial bleedings than warfarin, contemporary data showed that a considerable proportion of patients who meet the criteria for anticoagulant therapy are not treated and that rates of compliance with treatment recommendations are as low as 50% [20,21]. Moreover, some studies evaluating the benefit of educational intervention in stroke prevention did not show conclusive evidence in support of such an approach [22,23].

The main objective of this registry on AFIB is to describe key features of AFIB patients; other objectives include the analysis of contemporary patterns in AFIB management, the evaluation of major gaps in the guidelines’ [14,15,24] implementation in clinical practice and the correlation between management of AFIB and clinical outcomes, and the evaluation of the effectiveness of a web-based risk appraisal tool for the promotion of oral anticoagulation use. Our network database has the potential to provide a detailed evaluation of the key features of AFIB patients.

The present analysis of initial antithrombotic treatment from IN-AF registry strengthens some concepts in the context of anticoagulation therapy. First, the present analysis highlights that the estimated risk of bleeding strongly influences clinical decisions about use of anticoagulants in patients with AFIB. Each increment in HAS-BLED score was associated with lower probability of anticoagulation even after correction for embolic risk (OR: 0.62, 95% CI: 0.55 to 0.71, *p* < 0.0001).

Furthermore, 86% of patients with previous history of AFIB are discharged with a prescription for OACs, according to current guidelines [14,15,24]. Of these, only 69% received OACs at the baseline visit. Of note, rates of OACs prescription significantly increases in all the categories of baseline stroke risk (all *p* < 0.0001). Absolute differences are 16%, 18%, 19%, 12%, 11%, and 12% for CHA_2_DS_2_-VASc scores of 0, 1, 2, 3, 4, and >4, respectively. Thus, it is reassuring to find that prescription of OACs in patients with guideline-based indication was significantly raised from before the visit to discharge, with a parallel reduction in the use of antiplatelet drugs.

Finally, a web-based approach with immediate and automated estimate of thromboembolic and bleeding risk has the potential to improve the adequacy of anticoagulation treatment adhering to current guidelines [14,15,24]. As aforementioned, at the end of the inclusion visit with a web-based electronic estimate of risk scores for stroke and bleeding, the proportion of patients with a prescription for OACs was significantly raised. Notably, time-trend analysis showed non-significant changes in the prescription of oral anticoagulants over time (ranging from 82% in year 2013 to 80% after year 2019, *p* = 0.181), reinforcing the notion that a web-based system may help clinicians to choose the appropriate strategy to reduce the risk of cardioembolic events, taking into account the hemorrhagic risk of individual patients.

### Limitations

Our Registry on AFIB is useful to evaluate changes in the prescription of OACs over time. Nonetheless, the long recruitment time of the study may be considered as a main limitation.

In the case of a lack of prescription of OACs in a patient who met guidelines-derived indications for OAC, the web-tool of our registry asks them to specify the reason for the lack of such prescriptions. Nonetheless, a small but non-negligible proportion of patients did not receive OACs when they were recommended. The lack of prescription was incorrectly driven by the occurrence of long-term persistent or permanent AFIB. Future analyses on larger samples are needed to fully clarify the reasons for and the clinical impact of this aspect.

Finally, data on types of OAC prescribed at the end of the visit and the concomitant use of other medications are not still available.

## Figures and Tables

**Table 1:** Baseline characteristics of AFIB patients included in the analysis.

Variable	Overall (*n* = 2470)	Men (*n* = 1374)	Women (*n* = 1096)	*p*
Age (years) *	77 (70–83)	76 (67–82)	79 (72–85)	<0.0001
Current smokers (%)	6.8	8.7	4.5	<0.0001
Diabetes (%)	19.2	20.1	18.1	0.212
Body mass index (kg/m^2^)	27.2 (4.9)	27.4 (4.5)	26.9 (5.3)	0.011
Office systolic BP (mmHg)	130 (120–140)	130 (120–140)	130 (120–140)	0.371
Office diastolic BP (mmHg)	80 (70–85)	80 (70–85)	80 (70–82)	0.155
Hemoglobin (g/dL)	13.3 (2.1)	13.8 (2.1)	12.6 (1.9)	<0.0001
Creatinine (mg/dL)	1.08 (0.61)	1.16 (0.62)	0.98 (0.57)	<0.0001
eGFR < 60 (%)	35.5	28.2	44.7	<0.0001
Serum glucose (mg/dL)	101 (89–119)	100 (90–119)	101 (88–120)	0.363
Total cholesterol (mg/dL)	170 (139–198)	167 (134–194)	175 (142–202)	0.0002
LDL cholesterol (mg/dL)	98 (75–121)	98 (74–118)	100 (77–123)	0.139
Types of AFIB (%)				
First diagnosed	16.4	14.7	18.6	0.060
Paroxysmal	17.3	16.5	18.3	for trend
Persistent	28.4	29.5	27.0	
Long-standing	2.1	2.1	2.0	
Permanent	35.8	37.2	34.1	
Previous events				
Myocardial infarction (%)	14.0	17.1	10.1	<0.0001
Pulmonary embolism (%)	1.8	1.2	2.6	0.010
Stroke (%)	12.0	11.4	12.8	0.384
Transient ischemic attack (%)	6.6	7.0	6.0	0.339
Heart failure (%)	22.1	21.4	23.0	0.334

* denotes variables without normal distribution (for these variables, we reported median and interquartile range; see methods). BP = blood pressire; eGFR = estimated glomerular filtration rate; lDL = low density lipoprotein.

## Data Availability

The data underlying this article cannot be shared publicly due to the privacy of the individuals that participated in the study. The data will be shared on reasonable request to the corresponding author.

## Appendix A

www.italiafa.eu

ITALY-AF Investigators (alphabetical order by town)

Non-profit Sponsor: Fondazione Umbra Cuore e Ipertensione—ONLUS.

Scientific Secretary: Fabio Angeli.

**Participating Centers**: Hospital of Ancona and University of Ancona, Department of Cardiology (A. Capucci, F. Guerra, G. Ciliberti); Hospital of Ascoli Piceno, Department of Cardiology (P. Marchese, F. Gennaro, G. Mazzotta); Hospital of Fabriano, Department of Cardiology (M. Politano, P. Scipione); Hospital of Amelia, Department of Cardiology (M.L. Suadoni, S. Bergonzini); Hospital of Assisi, Department of Medicine (S. Bistoni, M. Trottini, C. Fattori, E. Cristofari); Hospital of Branca, Department of Medicine (S. Radicchia, A.O. Cazzato, M. Morini); Hospital of Branca, Department of Cardiology (E. Capponi, D. Cosmi); Hospital of Branca, Department of Neurology (D. Giannandrea); Hospital of Città di Castello, Department of Neurology (S. Ricci, M.R. Condurso, L. Greco); Hospital of Città di Castello, Department of Cardiology (A. Murrone, A. Contine, L. Marinacci, K. Mboumi); Hospital of Castiglione del Lago (C. Dembech, N. Sacchi, M. Guerrieri); Hospital of Perugia and University of Perugia, Medicina Interna e Vascolare (G. Agnelli, M.G. De Natale, C. Becattini, M.C. Vedovati); Hospital of Perugia and University of Perugia, Cardiologia e Fisiopatologia Cardiovascolare (G. Ambrosio, S. Sforna, C. Scapicchi, G. Giuffrè); Hospital of Perugia and University of Perugia, Medicina Interna (M. Pirro, V. Bianconi, M.R. Mannarino); Hospital of Perugia, Struttura Complessa di Cardiologia (C. Cavallini, G.L. Zingarini, F. Notaristefano, S. Coiro, C. Riccini, M. Dammando, A. Aita); Hospital of Perugia, Struttura Complessa di Pronto Soccorso (P. Groff, P. Cianci, S. Berisha); Hospital of Foligno, Struttura Complessa di Cardiologia (G. Bagliani, C. Andreoli, C. Mangialasche); Hospital of Orvieto, Struttura Complessa di Cardiologia (R. Di Cristofaro); USL Umbria 1, Cardiologia Ambulatoriale 1 (M.G. Pinzagli); USL Umbria 2, Cardiologia Ambulatoriale 2 (L. Filippucci, A. Faleburle); USL Umbria 2, Cardiologia Ambulatoriale (S. Repaci), USL Umbria 2, Cardiologia Ambulatoriale (G. Proietti); Hospital Media Valle del Tevere, Struttura Complessa di Medicina (U. Paliani, C. Fuoco, M.G. Conti, A. Cardona, C. Bartolini); Hospital of Terni, Struttura Complessa di Cardiologia (G. Carreras, C. Poltronieri, G. Khoury, A. Tordini); Hospital of Terni, Medicina Interna (G. Vaudo, G. Pucci, L. Sanesi, R. Curcio).

**Steering Committee**: Giancarlo Agnelli, Giuseppe Ambrosio^c^, Fabio Angeli, Antonio Capucci, Giovanni Carreras, Claudio Cavallini, Adriano Murrone, Gianpaolo Reboldi, Gaetano Vaudo, Paolo Verdecchia (Chairman), Gianluca Zingarini.
